# Clinical parameters of ovarian hyperstimulation syndrome following different hormonal triggers of oocyte maturation in IVF treatment

**DOI:** 10.1111/cen.13569

**Published:** 2018-03-06

**Authors:** A. Abbara, R. Islam, S.A. Clarke, L. Jeffers, G. Christopoulos, A.N. Comninos, R. Salim, S.A. Lavery, T.N.L. Vuong, P. Humaidan, T.W. Kelsey, G.H. Trew, W.S. Dhillo

**Affiliations:** ^1^ Hammersmith Hospital Imperial College London London UK; ^2^ IVF Unit Hammersmith Hospital London UK; ^3^ University of Medicine and Pharmacy at Ho Chi Minh City Ho Chi Minh City Vietnam; ^4^ My Duc Hospital IVFMD Ho Chi Minh City Vietnam; ^5^ The Fertility Clinic Skive Regional Hospital and Faculty of Health Aarhus University Aarhus Denmark; ^6^ School of Computer Science University of St Andrews St Andrews UK

**Keywords:** GnRH agonist, hCG, IVF, kisspeptin, ovarian hyperstimulation syndrome

## Abstract

**Objective:**

Ovarian hyperstimulation syndrome (OHSS) is a serious iatrogenic condition, predominantly related to the hormone used to induce oocyte maturation during IVF treatment. Kisspeptin is a hypothalamic neuropeptide that has recently been demonstrated to safely trigger final oocyte maturation during IVF treatment even in women at high risk of OHSS. However, to date, the safety of kisspeptin has not been compared to current hormonal triggers of oocyte maturation.

**Design:**

We conducted a retrospective single‐centre cohort study investigating symptoms and clinical parameters of early OHSS in women at high risk of OHSS (antral follicle count or total number of follicles on day of trigger ≥23) triggered with human chorionic gonadotrophin (hCG) (n = 40), GnRH agonist (GnRHa; n = 99) or kisspeptin (n = 122) at Hammersmith Hospital IVF unit, London, UK (2013‐2016).

**Results:**

*Clinical Parameters of OHSS*: Median ovarian volume was larger following hCG (138 ml) than GnRHa (73 ml; *P* < .0001), and in turn kisspeptin (44 ml; *P* < .0001). Median ovarian volume remained enlarged 20‐fold following hCG, 8‐fold following GnRHa and 5‐fold following kisspeptin compared to prestimulation ovarian volumes. Mean (±SD) ascitic volumes were lesser following GnRHa (9 ± 44 ml) and kisspeptin (5 ± 8 ml) than hCG (62 ± 84 ml; *P* < .0001). Symptoms of OHSS were most frequent following hCG and least frequent following kisspeptin. *Diagnosis of OHSS:* The odds ratio for OHSS diagnosis was 33.6 (CI 12.6‐89.5) following hCG and 3.6 (CI 1.8‐7.1) following GnRHa, when compared to kisspeptin.

**Conclusion:**

Triggering oocyte maturation by inducing endogenous gonadotrophin release is preferable to the use of exogenous hCG in women at high risk of OHSS.

## 1 INTRODUCTION

Ovarian hyperstimulation syndrome (OHSS) is a largely iatrogenic condition that is associated with significant morbidity and even mortality in otherwise healthy women undergoing fertility treatment.^1^ The hormone used to induce oocyte maturation plays a critical role in the risk of OHSS following IVF treatment, with exogenous human chorionic gonadotrophin (hCG) being the most frequent aetiological agent.^1^ Ovarian hyperstimulation syndrome results from the release of vasoactive substances from the ovary, such as vascular endothelial growth factor (VEGF A) due to excessive ovarian stimulation after triggering oocyte maturation during IVF treatment. This leads to increased capillary permeability and fluid shifts from the intravascular compartment into the third space of the body. Thus, OHSS is characterised by ovarian enlargement, ascites, pleural effusion, oliguria, haemoconcentration and thromboembolic events.^2^, ^3^

Ovarian hyperstimulation syndrome can be further categorised as early (≤9 days following trigger administration) or late (≥10 days) OHSS, to reflect differences in pathophysiology.^4^ Early OHSS results from excessive stimulation of the ovaries, usually due to the administration of hCG to trigger oocyte maturation.^4^ By contrast, late OHSS occurs due to the production of endogenous hCG from a developing pregnancy.^4^ Consequently, early OHSS is regarded as predominantly trigger‐related, whereas late OHSS is regarded as predominantly pregnancy‐related. Cryopreservation of embryos with transfer in later cycles (segmentation) can be used to manage the risk of OHSS, although logically, segmentation can only mitigate the occurrence of late OHSS.^5^ The risk of early OHSS can more reliably be mitigated using alternative triggers of oocyte maturation to hCG. Even so, 20% of IVF cycles started in Europe^6^ and 28% of IVF cycles conducted in the US^7^ necessitated segmentation to mitigate the risk of late OHSS.

Whilst hCG is the most commonly used trigger of oocyte maturation, it is also the most frequent aetiological factor in the development of OHSS, due to its long duration of action (7‐10 days). In women at high risk of OHSS, gonadotrophin‐releasing hormone agonist (GnRHa), which has a shorter duration of action than hCG, can be used as an alternative trigger of oocyte maturation. More recently, kisspeptin has been evaluated as a novel trigger of oocyte maturation.^8^ Kisspeptin acts to stimulate the release of endogenous GnRH from the hypothalamus. The peak serum LH level following kisspeptin occurs at a similar interval to GnRHa (~4 hours following administration), but to a lower amplitude, as kisspeptin only stimulates the release of an endogenous pool of GnRH.^9^, ^10^, ^11^ To date, there has been no direct comparison of markers of early OHSS following these triggers of oocyte maturation.

Several classifications have been proposed for the diagnosis of OHSS based on symptoms, sonographic and laboratory parameters. The most widely used criteria are those devised by Golan et al in 1989 with the updated classification by Navot et al in 1992.^2^, ^3^ However, due to a lack of objective normative data at OHSS screening, there has been a lack of consistency in the criteria used to diagnose OHSS. This has led to the formation of an “OHSS working group” who have recently published an expert consensus statement on the definition and the classification of OHSS in clinical trials.^12^ Most classifications diagnose mild OHSS if symptoms alone are present with mild ovarian enlargement (ovarian diameter 5‐12 cm), moderate OHSS if ascites on ultrasound is additionally present and severe OHSS if marked clinically apparent ascites (grade 2), pleural effusions, biochemical evidence of haemoconcentration or intravascular depletion are also evident.^2^, ^3^, ^12^

Whilst severe OHSS occurs in 2%‐6% of patients in an unselected population,^1^, ^13^ certain patient groups, such as those with polycystic ovaries, are at five‐fold increased risk of OHSS.^14^ Patients with at least 23 antral follicles have a four‐fold increased risk of clinically significant OHSS^15^ and studies in women with polycystic ovaries report rates of severe OHSS of ~11%‐16%.^13^, ^16^, ^17^

Therefore, we sought to characterise clinical parameters of OHSS (ovarian volume, ascitic volume) and symptoms of OHSS following different triggers of oocyte maturation (hCG, GnRHa or kisspeptin) in women at high risk of early OHSS (antral follicle count or total number of follicles on day of trigger ≥23).

## 2 METHODS

### 2.1 Study approval

Data from patients who received kisspeptin to trigger oocyte maturation were obtained from randomised clinical trials approved by the Hammersmith and Queen Charlotte's Research Ethics Committee, London, UK (reference: 10/H0707/2).^8^, ^10^, ^18^ The trials were registered on the National Institutes of Health Clinical Trials database (NCT01667406) and performed in accordance with the Declaration of Helsinki. All patients included in the study were treated at the IVF Unit at Hammersmith Hospital, London, UK, under a licence from the UK Human Fertilization and Embryology Authority.

### 2.2 IVF stimulation protocol

The protocol for patients undergoing kisspeptin triggering has been previously described.^8^, ^10^, ^18^ Patients triggered with hCG underwent either a GnRH agonist IVF protocol (long protocol), or a GnRH antagonist IVF protocol (short protocol), whereas all patients triggered with GnRHa or kisspeptin underwent the short protocol. Recombinant FSH (112.5‐250 IU Gonal F, Merck Serono, Geneva, Switzerland) was used to induce follicular growth in both long and short protocols. Premature ovulation was prevented by pretreatment with the GnRH agonist buserelin (0.2‐0.5 mg Suprefact, Sanofi‐Aventis, Gentily, France) in the long protocol, and with a GnRH antagonist—either cetrorelix (0.25 mg, Cetrotide, Merck Serono, Middlesex, UK) or ganirelix (0.25 mg Orgalutran, Merck Sharp & Dohme Ltd, Hertfordshire, UK) in the short protocol. The trigger was administered once 3 ovarian follicles were ≥18 mm in diameter and oocyte retrieval was conducted 36 hours thereafter. The trigger of oocyte maturation was either hCG—choriogonadotrophin alpha (0.25 mg, Ovitrelle; Merck Serono, Feltham, UK), GnRHa—buserelin acetate (2 mg, Suprecur, Sanofi‐Aventis, Guildford, UK), or kisspeptin‐54 (6.4‐12.8 nmol/kg as a single bolus, or 19.2 nmol/kg as a split dosing over 10 hours, Bachem Holding AG, Bubendorf, Switzerland) administered subcutaneously.^8^, ^10^, ^18^

### 2.3 Study participants

Women undergoing IVF treatment for infertility at Hammersmith Hospital, London, UK, between 2013 and 2016 were included in the study (n = 261). We selected patients at high risk of OHSS as defined by a total antral follicle count (AFC) or total follicle count on day of trigger ≥23.^15^

### 2.4 Assessment of early OHSS

Patients were assessed for parameters of early OHSS between days 2 and 6 following oocyte retrieval. For hCG and GnRHa groups, the Hammersmith IVF Unit policy is to screen all patients with at least 18 follicles ≥11 mm on the day of trigger for OHSS (all patients had follicle counts ≥23 on day of trigger in this study). Patients triggered with kisspeptin at high risk of OHSS (AFC ≥23) were part of a clincial trial and thus also screened for OHSS. In all patients, OHSS screening is usually conducted just prior to embryo transfer (either day 3 or day 5 postoocyte retrieval), although it may be conducted between days 2 and 6 following oocyte retrieval to avoid out‐of‐hours scheduling. During OHSS screening, patients were asked as to the presence of the following symptoms consistent with OHSS (abdominal pain, abdominal bloating, nausea, vomiting, diarrhoea, self‐reported reduction in urine output) and underwent transvaginal ultrasound screening to record parameters of OHSS such as ovarian size and the presence of ascites. Ovarian volumes were measured using transvaginal ultrasound (Toshiba Xario Prime, Crawley, UK) by experienced IVF physicians/ultrasonographers at Hammersmith IVF unit, London, UK during 2013‐2016 and converted electronically to ovarian volume values using the prolate ellipsoid formula (height × width × depth × 0.523). Patients were objectively graded for OHSS based on modified criteria of Golan et al 1989^2^ as below: *Normal*—Patients without any symptoms consistent with OHSS. *Mild OHSS*—Patients with at least one symptom consistent with OHSS and maximal ovarian volume (either left or right ovary) of between 65 and 903 ml (approximated to 5‐12 cm diameter). *Moderate OHSS*—Patients met the criteria for mild OHSS and additionally had at least 50 ml of total ascitic fluid in the pouch of Douglas, adnexae and abdomen. *Severe OHSS*—If patients met the above criteria for moderate OHSS, but additionally had at least 50 ml of fluid in the pleural space.

### 2.5 Statistical analysis

Statistical analysis was conducted by TWK and AA using Graphpad Prism version 7.0 and Stata version 14.0 statistical software. Baseline characteristics were compared using the Kruskal‐Wallis test for continuous variables and the Chi squared test for binary variables. Adjusted logistic regression analysis was used to derive odds ratios for experiencing symptoms of OHSS following hCG or GnRHa when compared to kisspeptin. Variables examined as potential confounders included age, body mass index (BMI), AFC, recombinant FSH dose, use of long IVF protocol, number of follicles ≥11 mm on day of trigger and number of oocytes retrieved. Univariate analyses were conducted to determine variables independently associated with both trigger and symptom and adjusted for in the logistic regression model. Odd ratios for abdominal pain and “self‐reported reduction in urine output” were adjusted for “use of long IVF protocol.” Odd ratios for abdominal bloating were adjusted for “use of long IVF protocol” and “number of oocytes” retrieved. Odd ratios for diarrhoea were adjusted for AFC and “use of long IVF protocol.” Odds ratios for nausea were adjusted for AFC, “total dose of recombinant FSH during stimulation” and “use of long IVF protocol.” No confounders significantly influenced the likelihood of diagnosis of OHSS and thus unadjusted odds ratios are presented. As no patient with kisspeptin triggering had “self‐reported reduction in urine output” or moderate OHSS, Laplace smoothing was applied.

## 3 RESULTS

### 3.1 Baseline characteristics

Two hundred and sixty‐one women at high risk of OHSS received either hCG (n = 40), GnRHa (n = 99), or kisspeptin (n = 122) to trigger oocyte maturation. A further 16 patients in the hCG group, 24 patients in the GnRHa group and 3 patients in the kisspeptin groups were identified who met the criteria for inclusion in the study, but did not have data available and were thus excluded from further analyses. The cohort studied was at high risk of OHSS with a median of 38 antral follicles, 24 follicles ≥11 mm on the day of trigger and 19 oocytes retrieved. There were significant imbalances between the treatment groups as clinicians are more likely to recommend GnRHa trigger rather than hCG trigger for patients deemed at higher risk of OHSS (“Table S1” for baseline characteristics).

### 3.2 Ovarian volume and ascitic volume following different triggers

**Figure 1:**
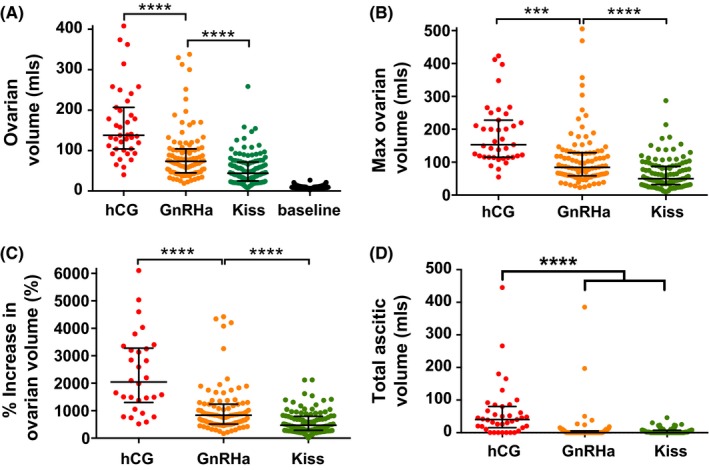
Clinical parameters of early ovarian hyperstimulation syndrome (OHSS) by trigger of oocyte maturation administered. A, Scattergram (median ± IQR) of mean ovarian volume at 2‐6 d following oocyte retrieval during IVF treatment in patients triggered with either human chorionic gonadotrophin (hCG) (n = 40), GnRH agonist (GnRHa; n = 99), or kisspeptin (n = 122). Baseline prestimulation median ovarian volume before stimulation with any of the 3 triggers is also presented. The three trigger groups were compared by the Kruskal‐Wallis test with post hoc Dunn's correction for multiple comparisons. ****P < .0001. B, Scattergram (median ± IQR) of maximal ovarian volume (largest ovarian volume of either ovary) at 2‐6 d following oocyte retrieval during IVF treatment in patients triggered with either hCG (n = 40), GnRH agonist (GnRHa; n = 99), or kisspeptin (n = 122). Groups were compared by the Kruskal‐Wallis test with post hoc Dunn's correction for multiple comparisons. ***P < .001, ****P < .0001. C, Scattergram (median ± IQR) of percentage increase in mean ovarian volume at 2‐6 d following oocyte retrieval when compared to prestimulation baseline values before IVF treatment in patients triggered with either hCG (n = 30), GnRH agonist (GnRHa; n = 90), or kisspeptin (n = 122). Groups were compared by the Kruskal‐Wallis test with post hoc Dunn's correction for multiple comparisons. ****P < .0001. D, Scattergram of total ascitic volume (median ± IQR) (free fluid in pouch of Douglas, adnexae and abdomen) at 2‐6 d following oocyte retrieval during IVF treatment in patients triggered with either hCG (n = 40), GnRH agonist (GnRHa; n = 99), or kisspeptin (n = 122). Groups were compared by Kruskal‐Wallis test with post hoc Dunn's correction for multiple comparisons. ****P < .0001

Median ovarian volume at 2‐6 days following oocyte retrieval was higher following hCG (138 ml) than following GnRHa (73 ml; *P* < .0001), than following kisspeptin (44 ml; *P* < .001) (Figure 1A). A multivariate regression analysis adjusted for AFC, body mass index, use of long IVF protocol, average daily recombinant FSH dose, number of follicles ≥11 mm on day of trigger and number of oocytes retrieved revealed an increase in mean ovarian volume of 151 ml (CI 113‐189; *P* < .001) following hCG and 29 ml (CI 8‐50; *P* = .007) following GnRHa when compared to kisspeptin. Mean total ascitic volume was 62 ml following hCG, 9 ml following GnRHa and 5 ml following kisspeptin (Figure 1D). A multivariate regression analysis adjusted for the same factors as above found that hCG resulted in an increase of 33 ml (CI 14‐52 ml; *P* = .001) of ascitic fluid when compared to kisspeptin.

### 3.3 Symptoms of OHSS following each trigger

**Figure 2:**
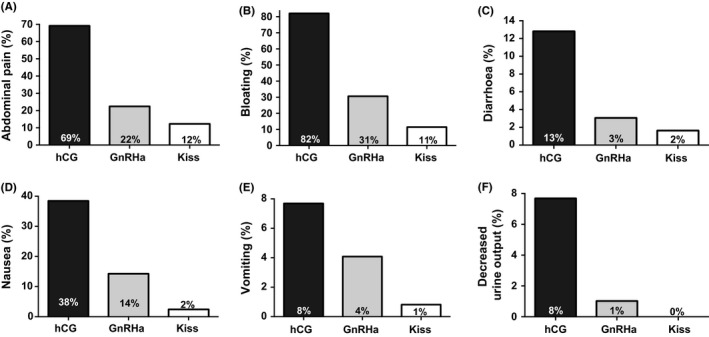
Symptoms of early ovarian hyperstimulation syndrome (OHSS) by trigger of oocyte maturation administered. The frequency of symptoms of OHSS by trigger of oocyte maturation is presented for patients receiving either human chorionic gonadotrophin (hCG) (n = 40), GnRH agonist (GnRHa; n = 99), or kisspeptin (n = 122). The frequency of symptoms consistent with OHSS including abdominal pain (A), abdominal bloating (B), diarrhoea (C), nausea (D), vomiting (E) and self‐reported reduction in urine output (F) are presented. The odds ratio of experiencing each symptom by trigger of oocyte maturation is presented in Table 1

Symptoms of OHSS were most frequent following hCG and least frequent following kisspeptin (Figure 2). Abdominal pain was ~13‐fold more likely following hCG (*P* < .001), and ~2‐fold more likely following GnRHa (*P* = .048) when compared to kisspeptin (Table 1). Abdominal bloating was ~30‐fold more likely following hCG (*P* < .001), and ~4‐fold more likely following GnRHa (*P* = .005) when compared to kisspeptin (Table 1). Nausea was ~19‐fold more likely following hCG (*P* = .001), and ~8‐fold more likely following GnRHa (*P* = .007) when compared to kisspeptin (Table 1).

**Table 1:** Symptoms of early OHSS using different triggers

	Univariate analyses		Multivariate analyses	
	Odds ratio (CI)	*P* value	Adj odds ratio (CI)	*P* value
Abdominal pain^a^				
hCG	16.1 (6.7‐38.3)	<.001	13.1 (4.2‐40.6)	<.001
GnRHa	2.1 (1.01‐4.2)	.048	2.1 (1.01‐4.2)	.048
Abdominal bloating^b^				
hCG	35.3 (13.1‐94.8)	<.001	30.7 (7.0‐134.3)	<.001
GnRHa	3.4 (1.7‐6.9)	.001	3.7 (1.5‐9.4)	.005
Diarrhoea^c^				
hCG	8.8 (1.6‐47.5)	.011	2.9 (0.2‐35.8)	.41
GnRHa	1.9 (0.3‐11.6)	.489	2.4 (0.4‐14.9)	.35
Nausea^d^				
hCG	24.8 (6.7‐92.3)	<.001	19.4 (3.3‐115.4)	.001
GnRHa	6.6 (1.8‐23.7)	.004	8.2 (1.8‐37.6)	.007
Vomiting^e^				
hCG	10.1 (1.02‐99.9)	.048	10.1 (1.02‐99.9)	.048
GnRHa	5.1 (0.6‐46.8)	.15	5.1 (0.6‐46.8)	.15
Self‐reported reduction in urine output^a^				
hCG	13.8 (1.5‐127.8)^f^	.021	16.1 (1.4‐188.8)^f^	.027
GnRHa	2.5 (0.2‐28.2)^f^	.45	2.5 (0.2‐28.2)^f^	.45

Univariate and multivariate logistic regression analysis of associations between symptom of early OHSS following different triggers. The odds ratio of experiencing symptoms of OHSS following hCG or GnRH trigger when compared with kisspeptin trigger is presented as follows: ^a^adjusted for use of long IVF protocol, ^b^adjusted for use of long IVF protocol and number of oocytes retrieved, ^c^adjusted for antral follicle count and use of long IVF protocol, ^d^adjusted for antral follicle count, total dose of recombinant FSH during stimulation and use of long IVF protocol, ^e^no confounders identified during univariate analysis. ^f^As no patient with kisspeptin triggering had self‐reported reduction in urine output, Laplace smoothing was applied.

### 3.4 Rates of OHSS following each trigger

Mild OHSS occurred in 45% of patients following hCG, 30% following GnRHa and 12% following kisspeptin (Table 2). Moderate to severe OHSS occurred in 37.5% of patients following hCG, 3% following GnRHa and no patient following kisspeptin (Table 2). The likelihood of OHSS was increased at least ~33‐fold following hCG (*P* < .0001) and ~3‐fold following GnRHa (*P* < .0001) when compared to kisspeptin (Table 2).

**Table 2:** Rates of early OHSS using different triggers

N	Normal	Mild OHSS	Moderate OHSS	Severe OHSS	Odds ratio of mild‐severe OHSS (95% CI)	Odds ratio of moderate‐severe OHSS (95% CI)
hCG (n = 40)	7 (18%)	18 (45%)	9 (23%)	6 (15.0%)	33.6 (12.6‐89.5) *P* < .0001	80.7^a^ (10.2‐637.5) *P* < .0001
GnRHa (n = 99)	66 (67%)	30 (30%)	3 (3%)	0 (0%)	3.6 (1.8‐7.1) *P* < .0001	5.1^a^ (0.6‐46.3) *P* = .15
Kisspeptin (n = 122)	107 (88%)	15 (12%)	0 (0%)	0 (0%)	‐	‐

The frequency (%) of the number of patients diagnosed with mild, moderate and severe OHSS is presented.

Patients were objectively graded based on modified criteria of Golan et al 1989^1^ as below:

*Normal*—Patients without any symptoms consistent with OHSS were graded as normal.

*Mild OHSS*—Patients with at least one symptom consistent with OHSS and maximal ovarian volume (either left or right ovary) of 65‐903 ml (approximated to 5‐12 cm diameter) were graded as mild OHSS.

*Moderate OHSS*—If patients met the above criteria for mild OHSS and additionally had at least 50 ml of total ascitic fluid in the pouch of Douglas, adnexae and abdomen, they were graded as *moderate OHSS*.

*Severe OHSS*—If patients met the above criteria for moderate OHSS, but additionally had at least 50 ml of fluid in the pleural space, they were graded as *severe OHSS*.

Rates of OHSS were compared by logistic regression: as no baseline variables were significant during univariate analyses, unadjusted odds ratios (95% confidence interval) are presented for hCG and GnRH agonist trigger as compared to kisspeptin trigger. Mild to severe OHSS was compared to “no OHSS,” whilst moderate‐severe OHSS was compared to “normal to mild OHSS.”

^a^As no patient with kisspeptin was diagnosed with moderate OHSS, Laplace smoothing was applied.

### 3.5 Ovarian volume as a marker of OHSS risk

Ovarian volume was larger in patients with more symptoms of OHSS and in patients with increased ascitic volume (Figure S1A‐B). Similarly, symptoms of OHSS and ascitic volume were increased in patients with larger ovarian volumes (Figure S1C‐D). No patient with a mean ovarian volume <100 ml was diagnosed with moderate to severe OHSS, whereas 16% of patients with mean ovarian volume between 101 and 200 ml and 56% of patients with a mean ovarian volume >200 ml were diagnosed with moderate to severe OHSS.

## 4 DISCUSSION

This study is the first to compare parameters of early OHSS following hCG, GnRHa and kisspeptin in women at high risk of OHSS. We observed that ovarian volumes following controlled ovarian stimulation recovered closer to baseline prestimulation volumes after GnRHa and kisspeptin than following hCG (ovarian volume was increased 20‐fold following hCG, 8‐fold following GnRHa, 5‐fold following kisspeptin; *P* < .0001) (Figure 1C). Furthermore, symptoms of OHSS were less frequent following GnRHa and kisspeptin than following hCG (Figure 2 and Table 1). The likelihood of diagnosis of OHSS was less frequent following the induction of endogenous gonadotrophins using GnRHa or kisspeptin than following exogenous hCG (Table 2).

Our results are consistent with the different mechanisms of action of the triggers used in the present cohort study. Accordingly, hCG acts directly on LH‐receptors in the ovary and has an excessive duration of action (7‐10 days) leading to an increased risk of OHSS.^1^ GnRHa has a significantly shorter duration of action than hCG and therefore carries a lower risk of inducing OHSS.^1^ Even so, recent case‐reports have suggested that GnRHa triggering can still result in severe early OHSS even in patients treated with segmentation and in the absence of hCG for luteal phase support.^19^, ^20^, ^21^, ^22^ Kisspeptin induces the release of an endogenous pool of GnRH from the hypothalamus, and thus results in the individualised release of gonadotrophins, which could be of particular value in patients at very high risk of OHSS.^10^, ^18^ Indeed, equimolar infusions of GnRH result in greater serum LH amplitude when compared to kisspeptin, implying that some pituitary GnRH receptors may not be directly contiguous with GnRH nerve terminals.^9^

Data from previous studies have suggested that the observed rates of OHSS following different triggers may not be entirely explained by their relative durations of stimulation and could additionally be due to direct ovarian actions. In this context, hCG has been shown to directly increase VEGF expression and raise VEGF levels in human granulosa cells to increase the risk of OHSS,^23^, ^24^, ^25^ whereas GnRHa may act directly on ovarian GnRH receptors to induce luteolysis to reduce the risk of OHSS.^26^, ^27^ Furthermore, the kisspeptin receptor has been hypothesised to play a key role in the pathogenesis of OHSS.^28^ Exogenous kisspeptin administration has been reported to reduce VEGF levels via a direct action on ovarian kisspeptin receptors to mitigate the risk of OHSS.^28^ This is in keeping with a recent clinical study comparing one or two doses of kisspeptin to trigger oocyte maturation, whereby a second dose of kisspeptin significantly increased the duration of the LH‐surge by 4‐10 hours in a cohort at high risk of OHSS, but did not result in increased ovarian volume or frequency of OHSS.^18^

Ovarian volume is dramatically enlarged following controlled ovarian stimulation in IVF treatment. The normal range for ovarian volume outside the context of IVF treatment in premenopausal women aged 23‐38 years is ~4‐10 ml.^29^ Ovarian volumes have been reported to be 73.4 cm^3^ at 5 days after oocyte retrieval following hCG trigger in the short IVF protocol (~9‐fold prestimulation volumes)^13^ and ~22 cm^3^ at 6 days after oocyte retrieval following a GnRHa trigger in women with normal ovarian reserve.^11^ Oyesanya and colleagues observed that mean ovarian volumes just prior to hCG trigger were larger in women who later developed moderate to severe OHSS (271 ml) when compared to women who did not develop OHSS (157 ml).^30^ These data are consistent with the higher ovarian volumes observed in the present study as our cohort was selected based on follicle count to be at high risk of OHSS.

Due to the frequency of segmentation in patients treated with hCG (51%) or GnRHa (13%) as compared to kisspeptin triggering (3%), it was not possible to reliably assess parameters of late OHSS. Whilst processess for vitrification of embryos have improved significantly in recent years, segmentation has been associated with an increased risk of pre‐eclampsia in women with polycystic ovarian syndrome.^31^ Enabling safe fresh embryo transfer through the avoidance of hCG trigger may facilitate a reduced “time to pregnancy,” which is often favoured by patients. Whilst the immediate welfare of otherwise healthy women undergoing fertility treatment is of paramount concern, OHSS occurrence may also increase the risk of late gestational complications such as prematurity and low birthweight.^32^, ^33^ Thus, there are a number of drivers to the use of safer triggers of oocyte maturation especially in the woman at high risk of OHSS.

Preventing premature ovulation during controlled ovarian stimulation prior to trigger using a GnRH agonist to downregulate the GnRH receptor (long IVF protocol) can be associated with a higher risk of OHSS than when this is achieved using a GnRH antagonist (short IVF protocol). Therefore, clinicians are more likely to treat patients at higher risk of OHSS with a GnRH agonist trigger in the short IVF protocol rather than to use an hCG trigger in the long IVF protocol. In patients triggered with hCG in the present study, 55% (22 of 40) had controlled ovarian stimulation using the long IVF protocol and 45% the short IVF protocol. Nevertheless, rates of moderate to severe OHSS were similar to those triggered with hCG in the context of the long protocol (40.9%) as in the short protocol (33.3%). Toftager and colleagues reported the frequency of OHSS symptoms at 5 days postoocyte retrieval using hCG trigger in the short IVF protocol in an unselected population to be: abdominal discomfort 67%, abdominal pain 44%, abdominal distension 31%, nausea 17%, vomiting 1% and shortness of breath 7%.^13^ Moreover, in the 11% of their patients with “irregular cycles” (implying the presence of polycystic ovarian syndrome), the rate of severe OHSS was increased almost 3‐fold at 11.1% (vs 4.4% in those with regular cycles) after hCG triggering in the short protocol.^13^ They observed that mean ovarian volume is ~29% higher and symptoms of OHSS 1%‐3% more frequent in patients triggered with hCG in the long protocol when compared to the short protocol.^13^ However, greater differences in ovarian volume and frequency of OHSS symptoms between the trigger cohorts were observed in the present study than could be attributed to differences in the stimulation protocol alone (median ovarian volume was increased by 214% following hCG and 66% following GnRHa compared to kisspeptin; ≥1 symptom of OHSS was present in 82% following hCG; 43% following GnRHa; 20% following kisspeptin). Additionally, the use of the long IVF protocol was statistically adjusted for when deriving odds ratios for parameters of OHSS. Thus, the published data are consistent with the high rates of symptoms observed in the present study given that this population was at increased risk of OHSS (mean number of oocytes retrieved in this cohort was ~20 as compared to ~9 in the Toftager cohort).^13^

All patients in this study were treated and assessed at the same centre and no more than 9 different IVF physicians/ultrasonographers conducted ultrasounds over the 3‐year study period. Limitations of the study include that insufficient data on blood results were available to assess for haemoconcentration and thus rates of severe OHSS may be underestimated. Indeed, patients in all three groups were at markedly increased risk of OHSS (median AFC ≥36 in all 3 groups) and the rate of severe OHSS encountered in the hCG group (15%) was consistent with the published literature for women with PCOS.^13^, ^16^, ^17^ All patients in the kisspeptin group, 96% of the GnRHa group and 75% of the hCG group had an antral follicle count ≥23, conferring an ~4‐fold increased risk of OHSS.^15^ Remaining patients with antral follicle counts <23 were included in the study (predominantly in the hCG group) if they had ≥23 follicles on the day of trigger (mean number of follicles on day of trigger was 35.9 in these patients suggesting that antral follicle count had been underestimated in these patients). However, this could have led to an underestimate of the difference between the trigger groups.

There were some differences in baseline characteristics between the treatment groups in this retrospective cohort study (Table S1); however, sensitivity analyses suggest that these were unlikely to have significantly altered the findings of this study. In 113 patients with at least 25 follicles ≥11 mm on day of trigger, mean ovarian volumes were (hCG 168.6, GnRHa 98.2, kisspeptin 66.9 ml), rates of mild to severe OHSS (hCG 87.5%, GnRHa 33.9%, kisspeptin 16%) and rates of moderate to severe OHSS were (hCG 37.5%, GnRHa 1.7%, kisspeptin 0%). In 114 patients with at least 20 oocytes retrieved, mean ovarian volumes were (hCG 176 ml, GnRHa 90.7 ml, kisspeptin 60 ml), rates of mild to severe OHSS (hCG 90.5%, GnRHa 32%, kisspeptin 10%) and rates of moderate to severe OHSS were (hCG 28.6%, GnRHa 2.7%, kisspeptin 0%). Whilst there was minor variation in the day of OHSS screening (≤0.6 days), 90% of assessments were conducted at 3‐5 days following oocyte retrieval and results were similar in 138 patients screened for OHSS only on day 5 following oocyte retrieval (mean ovarian volume: hCG 173.2, GnRHa 73.6, kisspeptin 50.1 ml). Although odds ratios were adjusted for relevant variables to account for differences in baseline characteristics, future prospective randomised clinical trials with objective grading of OHSS are required to confirm the results observed in this study.

In summary, we observe that the likelihood of clinical parameters of early OHSS was higher when triggering oocyte maturation with hCG than following GnRHa or kisspeptin. These results suggest that triggering oocyte maturation by the induction of endogenous gonadotrophin release is preferable to the use of hCG in women at high risk of OHSS.

## CONFLICT OF INTEREST

All authors declare that they have no competing interests.

## AUTHOR CONTRIBUTION

All authors provided contributions to study conception and design, acquisition of data or analysis and interpretation of data, drafting the article or revising it critically for important intellectual content, and final approval of the version to be published. Here are the most important contributions of each author: AA and WSD designed the study. Data were collected by AA, SC and RI. Analysis was carried out by TWK and AA. WSD takes final responsibility for this article.
