# Supplementary figures and images for: Clinical parameters of ovarian hyperstimulation syndrome following different hormonal triggers of oocyte maturation in IVF treatment
Source: Clin Endocrinol (Oxf). 2018 Mar 6;88(6):920–7. doi: 10.1111/cen.13569 (PMC6001461; doi:10.1111/cen.13569)

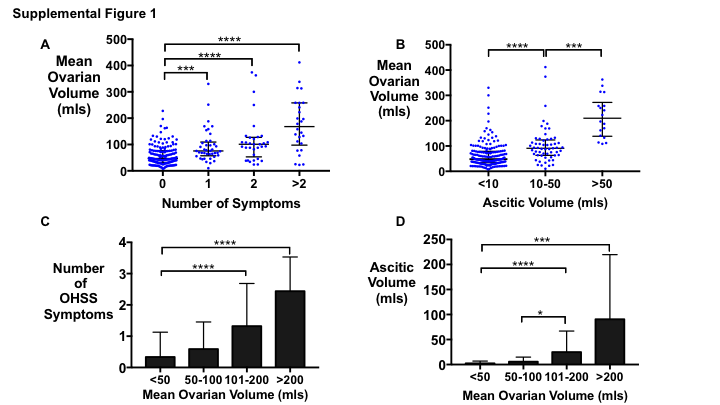

Supplement: Supplementary file 1 [file CEN-88-920-s001.tiff]
