# Supplementary material for: Clinical parameters of ovarian hyperstimulation syndrome following different hormonal triggers of oocyte maturation in IVF treatment
Source: Clin Endocrinol (Oxf). 2018 Mar 6;88(6):920–7. doi: 10.1111/cen.13569 (PMC6001461; doi:10.1111/cen.13569)
Supplement: Supplementary file 2 [file CEN-88-920-s002.docx]

**Supplemental Table 1: Baseline Characteristics**

|  | **hCG** | **GnRHa** | **Kisspeptin** | **All** | **P value** |
| --- | --- | --- | --- | --- | --- |
| **N** | **40** | **99** | **122** | **261** |  |
| **Age (y)** | 32·4  (30·0-35·3) | 30·0  (28·0-33·0) | 31·0  (29·0-33·0) | 31  (29-33) | 0·013 |
| **Body mass index (kg/m^2^)** | 24·6  (21·0-27·0) | 24·7  (21·0-27·7) | 24·5  (21·2-27·3) | 24·6  (21·0-27·3) | 0·97 |
| **Antral Follicle Count (AFC)** | 36  (20-40) | 42  (33-55) | 36  (29-49) | 38  (29-50) | <0·0001 |
| **Total recombinant FSH dose (IU)** | 1781  (1325-2325) | 1500  (1113-2025) | 1238  (1050-1575) | 1350  (1125-1950) | 0·0002 |
| **Mean daily rec FSH dose (IU)** | 150  (113-225) | 122  (111-150) | 113  (112-131) | 119  (113-150) | <0·0001 |
| **Long Protocol (%)** | 55% | 0% | 0% | 8% | <0·0001 |
| **Number of follicles**  **on day of trigger** | 36  (32-41) | 40  (35-48) | 33  (26-41) | 37  (29-34) | <0·0001 |
| **Number of follicles ≥11mm**  **on day of trigger** | 23  (20-31) | 27  (23-34) | 22  (15-27) | 24  (19-30) | <0·0001 |
| **Number of follicles ≥14mm**  **on day of trigger** | 16  (10-20) | 15  (13-21) | 13  (9-17) | 14  (10-19) | 0·001 |
| **Number of oocytes retrieved** | 21  (17-24) | 27  (20-34) | 12  (8-15) | 19  (12-27) | <0·0001 |
| **Mean day of OHSS assessment**  **(% between days 3-5**  **post oocyte retrieval)** | 4·1  (74%) | 4·1  (85%) | 4·7  (99%) | 4·4  (90%) | <0·0001 |
| **% treated with segmentation** | 51% | 13% | 3% | 17% | <0·0001 |

**Supplemental Table 1: Baseline characteristics**

Median (IQR) is presented for continuous variables and were compared by Kruskal Wallis test. Proportion is presented for binary variables and compared by Chi squared test. Long protocol denotes the use of a GnRH agonist to prevent premature ovulation during controlled ovarian stimulation prior to trigger.
